# Trends in Hospitalisations for Vaccine Preventable Respiratory Infections Following Emergency Department Presentations in New South Wales, Australia, 2012–2022

**DOI:** 10.1111/irv.70015

**Published:** 2024-09-26

**Authors:** Fariha Binte Hossain, David Muscatello, Sanjay Jayasinghe, Bette Liu

**Affiliations:** ^1^ School of Population Health UNSW Sydney Kensington New South Wales Australia; ^2^ National Centre for Immunisation Research and Surveillance (NCIRS), Kids Research, Sydney Children's Hospitals Network Westmead New South Wales Australia; ^3^ Children's Hospital at Westmead Clinical School The University of Sydney Sydney New South Wales Australia

**Keywords:** acute respiratory infections (ARI), emergency department (ED) presentations, hospitalisation trends, vaccine‐preventable respiratory infection

## Abstract

**Background:**

Vaccine‐preventable respiratory infections impact on healthcare systems globally. Despite availability of vaccines, fluctuations in vaccination rates, pathogen virulence and community transmission dynamics mean that these respiratory infections continue to pose substantial public health risks. To understand trends in vaccine‐preventable respiratory infections, we analysed linked data from emergency department (ED), hospitalisations and deaths in New South Wales, Australia, from 2012 to 2022.

**Methods:**

ED presentations with respiratory infection like illness were linked to hospitalisation and death records. Age‐standardised rates of ED presentations, proportions subsequently hospitalised for acute respiratory infection (ARI) and specific vaccine‐preventable disease diagnoses and 28‐day mortality rates were estimated by year and age.

**Results:**

From 2012 to 2022, there were 3,127,090 ARI‐like ED presentations. Age‐standardised rates increased until 2020, declined in 2021 and rebounded in 2022. Across all years, of these ARI‐like ED presentations, 16.6% were hospitalised for acute respiratory infections, including pneumonia (7.9%), influenza (1.1%), RSV disease (1.3%), COVID‐19 (0.8%) and pneumococcal disease (0.3%). Proportions hospitalised were highest in those aged 65+ years, except for RSV, which was highest in children aged 0–4 years. The highest 28‐day mortality post‐ARI‐like ED presentation was observed with COVID‐19 in adults aged 65+ years at 13.1%.

**Conclusions:**

This study highlights the continuing burden of vaccine‐preventable respiratory infections on an Australian healthcare system. These data can be used to monitor the effectiveness of vaccination programmes and other public health interventions. Future efforts should focus on enhancing surveillance and data linkage to improve precision and guide targeted public health strategies.

## 1 Introduction

The burden of vaccine‐preventable respiratory infections such as influenza, respiratory syncytial virus (RSV), COVID‐19 and pneumococcal disease, on healthcare systems globally, is substantial [1, 2, 3]. In 2021, a global meta‐analysis showed that influenza viruses caused over 5 million adult hospitalisations for lower respiratory tract infections (LRTIs) [4]. Another study reported that RSV caused 3.6 million hospitalisations among children under five in the same year [5]. In 2021, *Streptococcus pneumoniae* caused an estimated 505,000 deaths from LRTIs globally [6], and the COVID‐19 pandemic has been responsible for approximately 14.9 million deaths (ranging from 13.3 to 16.6 million) from 1 January 2020 to 31 December 2021 [7]. This pandemic further intensified challenges on healthcare systems worldwide [8, 9, 10]. Respiratory infections are a major public health concern in Australia, accounting for 6–7 million GP visits each year and ranked the sixth leading cause of death [9]. Despite availability of vaccines, fluctuations in vaccination rates, pathogen virulence and community transmission dynamics mean that these respiratory infections continue to pose substantial public health risks. Therefore, assessing the burden of these infections is crucial for planning and evaluating public health interventions, such as optimisation of vaccination strategies, and identifying healthcare strategy gaps.

In this study, we analysed linked administrative data covering emergency department (ED) presentations, hospital admissions and mortality records for New South Wales (NSW) residents from January 2012 to December 2022, which included the COVID‐19 pandemic period. The objectives of our study were to investigate trends in rates of ED presentations throughout the study period and to quantify the proportion of those presentations that resulted in hospital admission for potentially preventable vaccine‐preventable diseases and subsequent mortality.

## 2 Methods

### 2.1 Study Population and Datasets

We conducted a population‐based, retrospective analysis using linked administrative data covering the period from January 2012 through December 2022 in NSW, Australia's most populous state (population 8.2 million in 2022). Data were obtained from the pandemic and epidemic risk assessment using linked (PEARL) database. The database was prepared using privacy‐preserving, probabilistic record linkage [10] to allow research on short‐term (28 days) health outcomes for individuals presenting to an ED in a public hospital and assigned a diagnosis potentially relating to an acute respiratory infection (ARI). In the PEARL database, ED presentations from the NSW Emergency Department Data Collection (EDDC) are linked to the NSW Admitted Patient Data Collection (APDC) and deaths registered in the NSW Registry of Births Deaths & Marriages (RBDM). Data linkage was conducted by the NSW Centre for Health Record Linkage (https://www.cherel.org.au/) using a probabilistic method with a reported false positive linkage rate of at most 0.5% [8].

The EDDC contains administrative and clinical data from patients presenting to public hospital EDs in NSW [11]. Over time, the number of EDs included in the EDDC has increased, and in recent years, it has expanded to include almost all of the smaller ED facilities, representing 95% of all ED attendances in NSW [12]. For consistency in examining trends, out of 185 EDs reporting to the EDDC, we excluded 54 from our analysis if there were incomplete records in any year from 2012 to 2022. During this study period, the excluded hospitals accounted for a total of 104,255 ED admissions. ED data included age, sex, arrival date and discharge diagnoses coded using the International Classification of Diseases and Related Problems 9^th^ and 10^th^ Revisions, Australian Modification (ICD‐9‐AM, ICD‐10‐AM) or the Systematised Nomenclature for Medicine‐Clinical Terms (SNOMED‐CT).

The APDC has comprehensive data on all hospital admissions in NSW and includes demographic information, admission and separation dates and clinical diagnoses. Principal and up to 50 additional diagnoses contributing to the admission are recorded using the ICD‐10‐AM [13]. The RBDM data provide age and date of death.

The New South Wales Population Health and Health Services Research Ethics Committee granted ethics approval for this study (2021/ETH00070).

### 2.2 Outcome Definitions

We examined three outcomes. First, we looked at “ARI‐like” ED presentations defined by the selected symptoms or disease codes (using ICD 9/10 AM and SNOMED‐CT) provided in Table S1. Second, among those ARI‐like ED presentations, we examined the proportion hospitalised within 1 day of the ED presentation that had one of a selected number of respiratory diagnoses. These included ‘all‐ARI’, ‘all‐cause pneumonia’, ‘influenza’, ‘RSV disease’, ‘COVID‐19’ and ‘pneumococcal disease’ and were based on ICD‐10‐AM codes recorded in either the primary or additional diagnosis fields. The specific codes selected included were as follows: ARI (J00‐J22, U07.1, U07.2, B34.2, B97.2, B97.4), which includes subsets such as all‐cause pneumonia (J10.0, J11.0, J12‐J18) along with causative‐organism specific conditions such as influenza (J09‐J11), RSV disease (J12.1, J20.5, J21.0; B97.4), COVID‐19 (B34.2: Coronavirus infection, unspecified site; B97.2: Coronavirus as the cause of diseases classified to other chapters; U07.1: COVID‐19, virus identified; and U07.2: COVID‐19, virus not identified) and pneumococcal disease (J13, J18.1). For further details, see Table S2. Third, we assessed the 28‐day mortality following the ED arrival date among those hospitalised with one of the conditions listed above. For analyses of COVID‐19, we only used records from 2020 onwards.

### 2.3 Statistical Analysis

We described the characteristics of the study population presenting to ED with ARI diagnoses or symptoms overall and by year. We then estimated the monthly age‐standardised population rate of ARI‐like ED presentations during the study period by direct standardisation using the 2016 NSW midyear population estimates from the Australian Bureau of Statistics (ABS) as the standard population [14]. For each calendar month, we calculated the ED visit rate per 100,000 population overall and by age groups: 0–4, 5–14, 15–39, 40–64 and 65+ years.

Next, we calculated the annual proportion of ARI‐like ED presentations hospitalised for ARI within one day of ED arrival. The hospitalisation proportions were calculated for the six conditions. The numerator for each proportion was the number of hospital admissions with a relevant diagnosis, and the denominator was the total number of included ARI‐like ED presentations. We presented these proportions for the overall population and by age group. Finally, we calculated the 28‐day mortality rate for each age group among patients hospitalised for ARI, all‐cause pneumonia, influenza, RSV disease, COVID‐19 and pneumococcal disease. For each category, the 28‐day mortality is the proportion of hospitalised patients who died within 28 days of ARI‐like ED presentations.

The region of residence for study participants was determined based on the Statistical Area Level 2 (SA2) recorded in the EDDC, as classified using the Australian Statistical Geography Standard (ASGS) Edition 2016. For the purposes of this study, regions were categorised into ‘Greater Sydney’ and ‘Rest of NSW’ [15].

All analyses were performed using Stata 17.0 software (StataCorp, Texas, USA).

## 3 Results

### 3.1 Characteristics of the Study Population

During the study period, there were a total of 3,127,090 ARI‐like ED presentations among 2,525,148 persons at 131 included NSW public hospital EDs from 2012 to 2022. Table 1 provides the yearly distribution of characteristics of these patients. There was a substantial increase in the annual number of ARI‐like ED presentations from 228,705 in 2012 to 404,155 in 2022. Similarly, examining patients presenting to ED rather than number of presentations, we observed an increase from 2012 (*n* = 187,864) to 2017 (*n* = 244,707) with variations from 2017 to 2021 and a substantial increase in 2022 (*n* = 322,934). Of the total, in each year of the study period, 12.5% of individuals had two presentations and 4.4% had three or more presentations. From 2012 to 2022, the mean and median age of those presenting rose from 29.4 to 31.4 years, (median: from 18.4 to 23.0 years). Young children (aged 0–4 years) were the most frequent presenters (33.7%). Throughout the study period, males more frequently presented to ED than females, except in 2022, where the distribution was slightly skewed towards females (50.3%). Just over one half of the individuals presenting to the ED in our study were residents of Greater Sydney.

**TABLE 1:** Characteristics of the study population presenting with acute respiratory infections (ARI) or symptoms to the emergency department (ED) in NSW public hospitals by year, 2012–2022.

	Year											Total
	2012	2013	2014	2015	2016	2017	2018	2019	2020	2021	2022	
No. of ARI‐like ED presentations	228,705	234,214	253,578	272,993	272,460	303,483	278,001	335,145	270,588	273,768	404,155	3,127,090
No. of patients	187,864	190,702	205,047	219,990	219,792	244,707	221,611	266,900	224,382	221,219	322,934	2,525,148
Median age	18.4	18.0	18.1	18.4	19.9	21.3	20.0	20.3	25.9	22.6	23.0	
Mean age (±SD)	29.4 (29.9)	29.1 (29.9)	29.2 (30.1)	29.4 (29.9)	30.4 (30.4)	31.4 (30.4)	30.6 (30.5)	30.3 (29.9)	31.8 (28.9)	30.6 (29.5)	31.4 (29.8)	
Age groups												
0–4 years	65,812 (35.0)	68,595 (36.0)	74,914 (36.5)	76,092 (34.6)	75,894 (34.5)	79,726 (32.6)	79,061 (35.7)	88,449 (33.1)	67,780 (30.2)	76,120 (34.4)	99,412 (30.8)	851,855 (33.7)
5–14 years	22,524 (12.0)	22,369 (11.7)	23,079 (11.3)	27,812 (12.6)	24,778 (11.3)	29,343 (12.0)	23,129 (10.4)	33,426 (12.5)	21,751 (9.7)	20,192 (9.1)	39,101 (12.1)	287,504 (11.9)
15–39 years	37,089 (19.7)	36,596 (19.2)	38,982 (19.0)	43,312 (19.7)	42,952 (19.5)	47,482 (19.4)	41,476 (18.7)	54,379 (20.4)	53,702 (23.9)	48,011 (21.7)	69,697 (21.6)	513,678 (21.2)
40–64 years	27,062 (14.4)	27,718 (14.5)	29,194 (14.2)	31,299 (14.2)	32,076 (14.6)	37,106 (15.2)	32,574 (14.7)	38,388 (14.4)	39,904 (17.8)	35,441 (16.0)	50,345 (15.6)	371,107 (15.3)
65+ years	35,377 (18.8)	35,422 (18.6)	38,876 (19.0)	41,475 (18.9)	44,092 (20.1)	51,050 (20.9)	45,370 (20.5)	52,258 (19.6)	41,245 (18.4)	41,455 (18.7)	64,379 (19.9)	490,999 (20.3)
Sex												
Male	96,085 (51.2)	98,146 (51.5)	104,832 (51.1)	112,262 (51.0)	112,335 (51.1)	123,967 (50.7)	113,220 (51.1)	135,177 (50.7)	112,917 (50.3)	111,741 (50.5)	160,458 (49.7)	1,281,140 (52.8)
Female	91,767 (48.9)	92,522 (48.5)	100,210 (48.9)	107,730 (49.0)	107,453 (48.9)	120,735 (49.3)	108,384 (48.9)	131,717 (49.3)	111,449 (49.7)	109,459 (49.5)	162,450 (50.3)	1,243,876 (51.3)
No of visits/person												
One	158,206 (84.2)	159,627 (83.7)	170,915 (83.4)	182,609 (83.0)	182,411 (83.0)	203,140 (83.0)	182,495 (82.4)	219,454 (82.2)	190,514 (84.9)	183,642 (83.0)	266,115 (82.4)	2,099,128 (83.1)
Two	22,408 (12.0)	23,220 (12.2)	25,245 (12.3)	27,607 (12.6)	27,768 (12.6)	30,920 (12.6)	28,548 (12.9)	34,668 (13.0)	25,858 (11.5)	28,038 (12.7)	41,544 (12.9)	315,819 (12.5)
Three or more	7178 (3.8)	7855 (4.1)	8887 (4.3)	9778 (4.4)	9613 (4.3)	10,647 (4.4)	10,568 (4.7)	12,778 (4.8)	8010 (3.6)	9540 (4.3)	15,275 (4.7)	110,129 (4.4)
Reigon of residence												
Greater Sydney	97,354 (51.8)	101,702 (53.3)	108,813 (53.1)	118,097 (53.7)	115,160 (52.4)	127,392 (52.0)	117,505 (53.0)	143,454 (53.8)	122,845 (54.8)	117,026 (52.9)	175,726 (54.4)	1,345,070 (53.3)
Rest of NSW	84,806 (45.1)	83,045 (43.6)	89,812 (43.8)	95,192 (43.3)	97,489 (44.4)	109,338 (44.7)	96,611 (43.6)	114,845 (43.0)	95,882 (42.7)	99,389 (44.9)	138,425 (42.9)	1,104,834 (43.8)
Missing	5704 (3.0)	5955 (3.1)	6422 (3.1)	6705 (3.0)	7143 (3.2)	7977 (3.3)	7495 (3.4)	8601 (3.2)	5655 (2.5)	4805 (2.2)	8614 (2.7)	73,076 (2.9)

*Note:* Characteristics of the study population are based on counts of patients not presentations.

### 3.2 Monthly Age‐Standardised Rate of ARI‐Like ED Presentations 2012 to 2022

**FIGURE 1:**
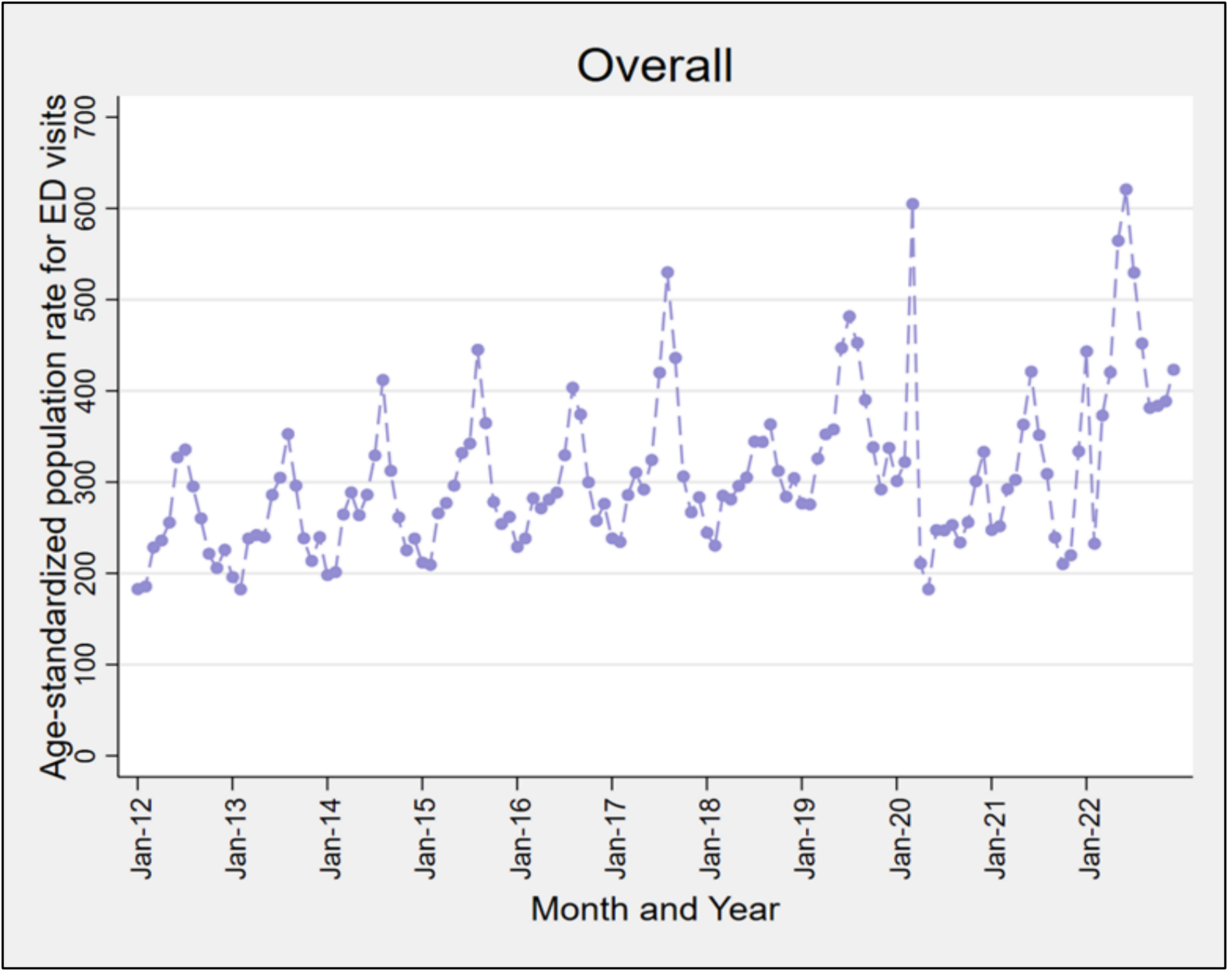
Age‐standardised rates for ARI‐like ED presentations symptoms per 100,000 population, by month, across 131 ED's in NSW, January 2012 to December 2022.

**FIGURE 2:**
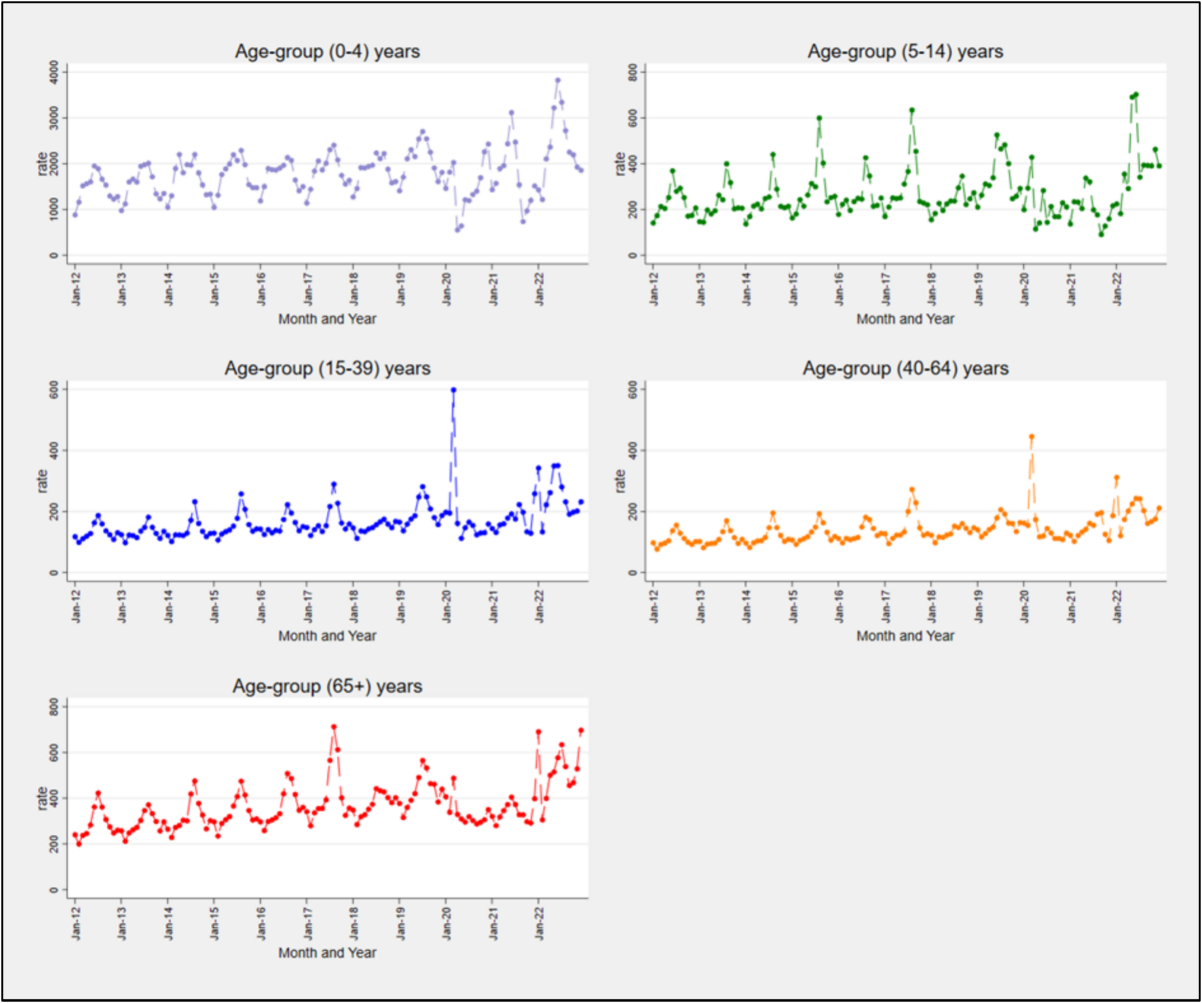
Age‐standardised rate for ARI‐like ED presentations per 100,000 population, by month and age group, across 131 ED's in NSW, January 2012 to December 2022.

From January 2012 to December 2022, we identified a total of 3.1 million ARI‐like ED presentations for respiratory infections or related symptoms, with annual peaks in monthly rates during June–September and troughs in January–February until 2019 (Figure 1). In 2020, a peak occurred in March (604 presentations per 100,000 population), then sharply declined. In 2021 and 2022, peaks returned in June, reaching 620 presentations per 100,000 in June 2022. Throughout the study period, the highest ED presentation rate was observed in children aged 0–4 years, peaking in June 2022 at 3823 presentations per 100,000 (Figure 2). The 65+ years age group also experienced high ED presentation rates, with substantial peaks in July 2017 (712 presentations per 100,000), January 2022 (690 presentations per 100,000), July 2022 (635 presentations per 100,000) and December 2022 (698 presentations per 100,000). The 5‐ to 14‐year age group saw significant peaks in August 2017 (634 presentations per 100,000) and June 2022 (702 presentations per 100,000). However, the highest peaks for the 15–39 years and 40–64 years age groups occurred in March 2020 (598 and 445, respectively per 100,000 population) (Figure 2).

### 3.3 Hospitalisations Following ARI‐Like ED Presentations by ARI Category

Of the 3.1 million ARI‐like ED presentations, 519,145 (16.6%) were hospitalised with an ARI within 1 day of their ED presentation. Of these, 247,818 (7.9%) were for all‐cause pneumonia, 34,368 (1.1%) for influenza, 40,411 (1.3%) for RSV and 8815 (0.3%) for pneumococcal disease. Of the 948,520 ARI‐like ED presentations from 2020 onwards, 24,752 (2.6%) hospitalisations had a COVID‐19 diagnosis.

**FIGURE 3:**
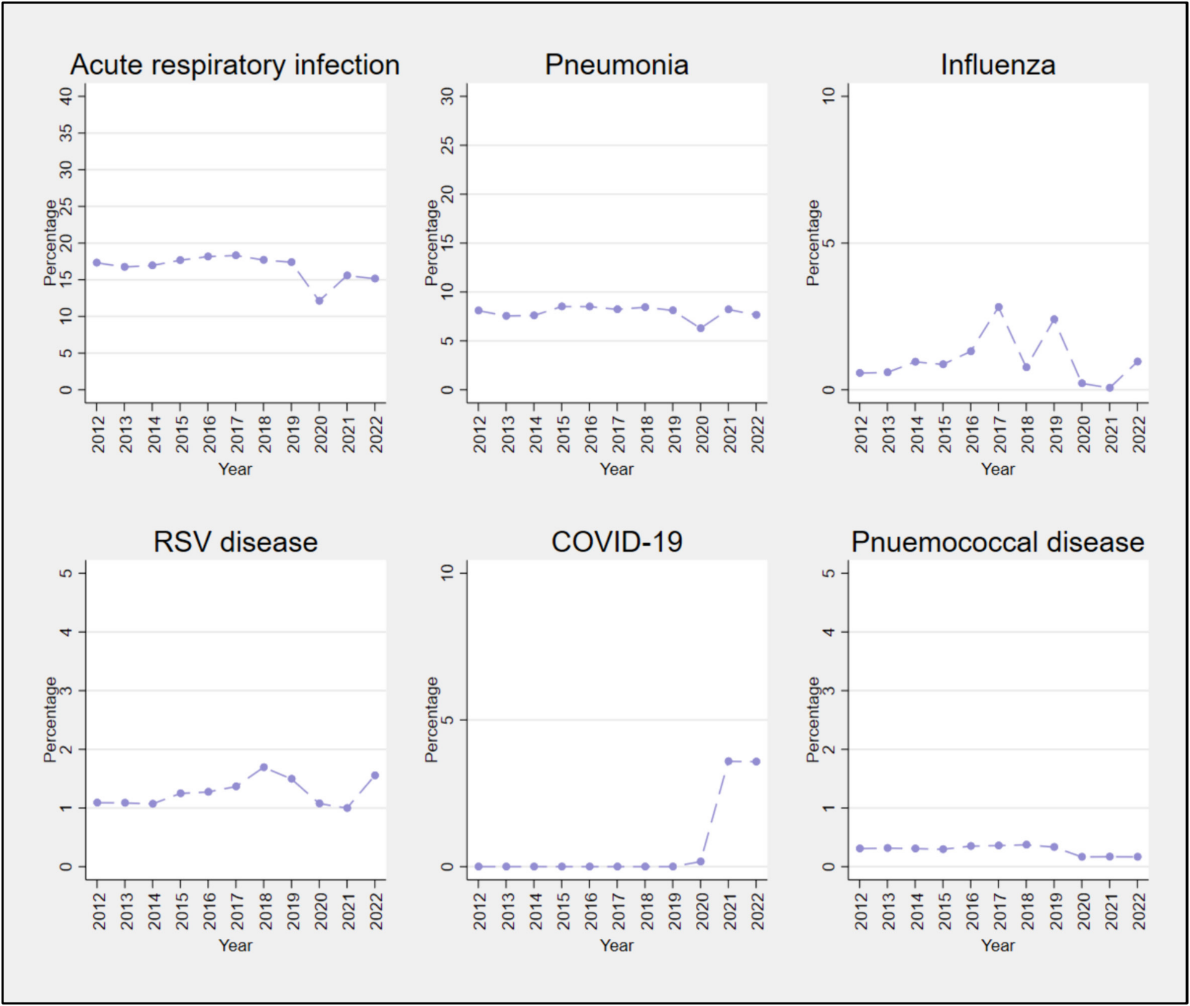
Percentage of ARI‐like ED presentations that were hospitalised with any admission diagnosis of ARI, all‐cause pneumonia, influenza, RSV disease, COVID‐19 or pneumococcal disease, by year, 2012 to 2022.

The annual proportion of ARI‐like ED presentations that were hospitalised for an ARI remained stable at around 17% until 2019, then decreased to 12% in 2020, and rose again to 15% in 2021 and 2022 (Figure 3). For the specified vaccine‐preventable conditions, hospitalisations declined in 2020, except for COVID‐19 which varied from 0.2% in 2020 to 3.6% in 2022 (Figure 3). There were two peaks in the observed proportion hospitalised for influenza: 2.8% in 2017 and 2.4% in 2019; for RSV, peaks were observed at 1.7% in 2018 and again at 1.6% in 2022.

**FIGURE 4:**
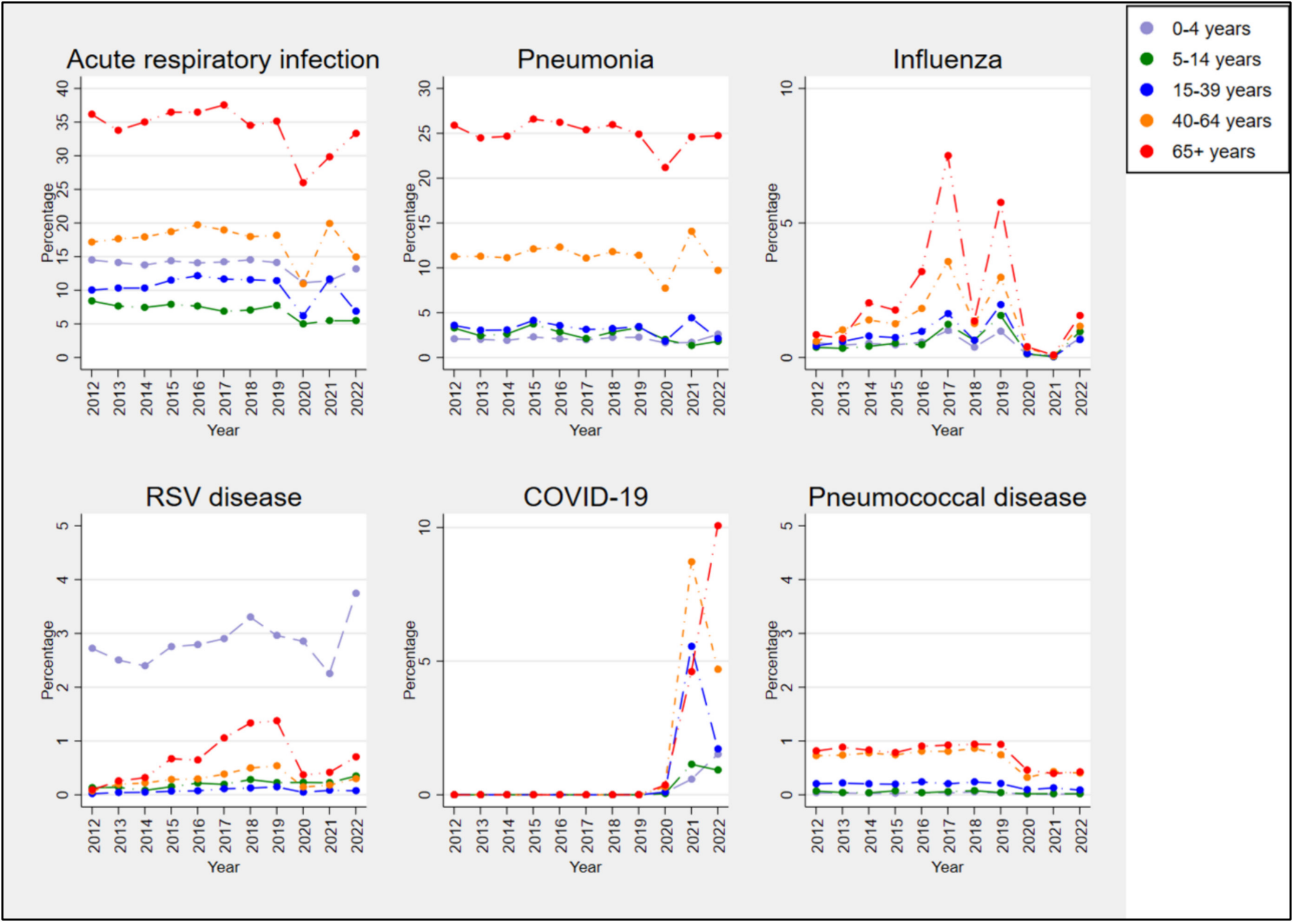
Percentage of ARI‐like ED presentations hospitalised with any admission diagnosis of ARI, all‐cause pneumonia, influenza, RSV disease, COVID‐19 and pneumococcal disease, by age group, from 2012 to 2022.

For all respiratory conditions examined, except RSV, the highest proportions hospitalised were among those aged 65+ years (see Figure 4). For RSV, hospitalisations were highest in 0–4 years old, with a rate of 2.7% in 2012 that rose to 3.7% by 2022. Pneumococcal disease hospitalisations in the 65+ age group were around 0.9% in 2012 to 2019 and decreased to 0.5% during 2020 to 2022. The highest proportion hospitalised for COVID‐19 was observed for patients aged 65+ years in 2022 (10.1%) (Figure 4).

### 3.4 28‐Day Mortality Rate Following ARI‐Like ED Presentations Among Those Who Were Hospitalised With Respiratory Infections

**FIGURE 5:**
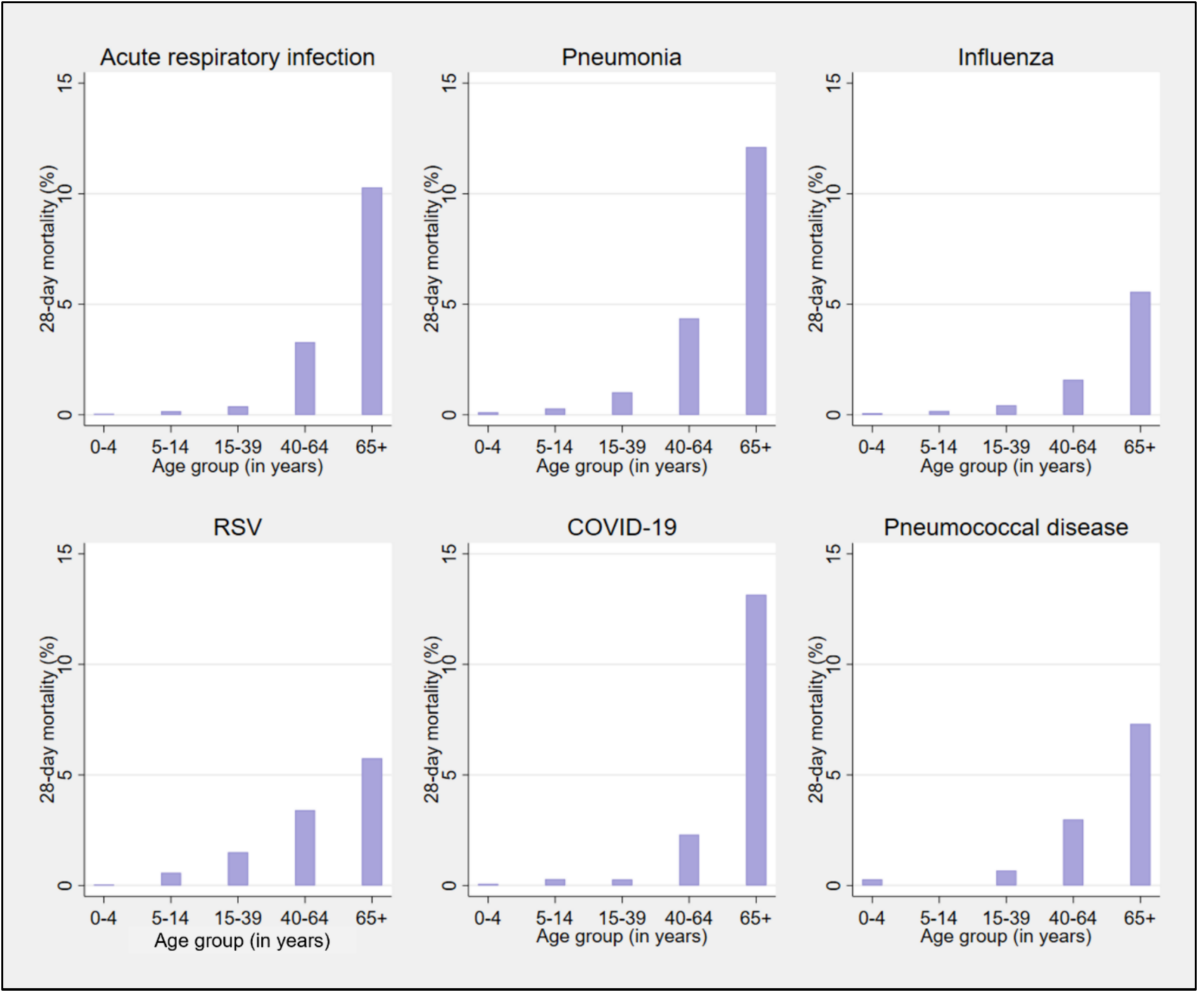
The 28‐day mortality rate following ARI‐like ED presentations among those who were hospitalised with acute respiratory infection (ARI), all‐cause pneumonia, influenza, RSV disease, COVID‐19 and pneumococcal disease in NSW, by age group, from 2012 to 2022.

Among individuals hospitalised for each condition, the number of deaths within 28 days following ARI‐like ED presentations was as follows: for ARI, 23426 (4.5%) out of 519,145; for all‐cause pneumonia, 20,155 (8.1%) out of 247,818; for influenza, 941 (2.7%) out of 34,368; for RSV disease, 305 (0.8%) out of 40,411; for COVID‐19, 1505 (6.1%) out of 24,752; and for pneumococcal disease, 414 (4.7%) out of 8815. Mortality increased with increasing age. For ARI, these rates ranged from 0.1% in the 0–4 years age group to 10.3% in those aged 65 years and above (Figure 5). Mortality data for each age group and category can be found in Table S3. This age‐related increase in mortality was consistent across all the vaccine‐preventable respiratory conditions examined. Among those aged 65+ years, those hospitalised for COVID‐19 following an ED presentation had the highest mortality rate at 13.1%, with all‐cause pneumonia following at 12.1% (see Figure 5).

## 4 Discussion

In this population‐based study involving ED, hospital admission and death registration data from 2012 to 2022, we identified a clear seasonal trend in rates of ARI‐like ED presentations, with annual peaks in June–September, except for the first year of the COVID‐19 pandemic (i.e., 2020) when the peak occurred in March. The highest population rate of ARI‐like ED presentations was among children aged 0–4 years, followed by those aged 65 years and older. We found that 16.6% of ARI‐like ED presentations were hospitalised for ARI within 1 day. The 65+ years age group had the highest hospitalisation rates following ED presentation for all respiratory conditions, except for RSV, which was highest in the 0–4 years age group. The 28‐day mortality rates following ARI‐like ED presentations among those who were hospitalised increased with increasing age. Patients over 65 years old, particularly those hospitalised for COVID‐19 following their ED presentation, had the highest mortality rates.

Our study highlighted peaks in ARI‐like ED presentations from June to September, aligning with Australia's winter and early spring. A previous study found that between 2010 and 2014, influenza contributed to more than one in ten ED visits related to respiratory or infectious conditions, with most occurring from midwinter to early spring [16]. Additionally, this seasonal pattern mirrors the trends observed in the Australian Influenza Surveillance Report and NSW influenza surveillance data, which also recorded increased notifications of influenza, fever and unspecified infections across all age groups during these months from 2015 to 2020 [17, 18]. In 2020, we observed a peak in March, followed by a marked decline, reflecting early pandemic healthcare seeking behaviours and the initial spread of COVID‐19 [17, 18]. The usual winter peak was absent, likely due to nonpharmaceutical interventions in place for mitigating COVID‐19 transmission and the related altered healthcare utilisation [19]. We observed a notable surge in ARI‐like ED presentations across most age groups in 2022, which was also reported by the Australian Institute of Health and Welfare (AIHW) [20]. This increase was likely driven by the lifting of COVID‐19 restrictions, the reopening of international borders and heightened community transmission of respiratory pathogens. Additionally, the rise in PCR and multiplex testing in 2022 may have contributed to the increase in case ascertainment [21]. Consistent with previous literature, we observed the highest rates of ARI‐like ED presentations among children aged 0–4 years, followed by adults aged 65 years and above [22, 23]. A recent study indicates that people over 65 account for up to 41% of ED presentations in Australia [23], with respiratory infections being a leading cause [24]. This highlights the potential impact of COVID‐19 restrictions: reducing immediate transmission but subsequent lowered immunity in vulnerable groups [25].

In our study, we found that males visited the ED more frequently than females throughout the study period, except in 2022 when the visits slightly favoured females (50.3%) which has also been found in a national report [26]. We also observed that just over half of the individuals presenting to the ED in our study were residents of Greater Sydney. Given that roughly 66% of the state's population lives in Greater Sydney [27], those presenting to ED with ARI‐like illnesses were underrepresented by the population distribution in the state.

We observed that despite an increase in ARI‐like ED presentations over the years, the hospitalisation rate for ARI following ED presentation remained consistent until the COVID‐19 pandemic disrupted this trend in 2020. This contrasts with earlier studies that reported a gradual increase in hospitalisation rates over time, followed by a notable decline in the first year of the COVID‐19 pandemic [24, 28]. However, these studies reported hospitalisation rates per population, without linking them to prior ED presentations. When we looked at the trend according to age groups and specific conditions, we observed that individuals aged 65 years and older consistently had the highest hospitalisation rates for all respiratory infections, except RSV. This older age group also exhibited the highest 28‐day mortality rate following ARI‐like ED presentations and hospitalisation, with the highest rate observed for COVID‐19.

Hospitalisations for influenza following ARI‐like ED presentations peaked in 2017 (2.8%) and 2019 (2.4%), coinciding with influenza epidemics in Australia. Previous studies reported increased rates of hospital admissions, ICU stays and mortality due to influenza during these periods [29, 30, 31, 32]. The outbreaks in 2017 and 2019 may be caused by a mismatch between the circulating influenza viruses, and the strains included in the annual flu vaccine with a previous study estimating vaccine effectiveness in 2017 was only 33% [33]. The patterns observed during the study period were likely influenced by changes in immunisation coverage, particularly during the COVID‐19 pandemic [34] and may have played a role in shaping these trends. For example, disruptions in routine vaccination schedules and fewer public health campaigns might have led to lower vaccination rates. This drop in coverage may have temporarily reduced immunity in some groups, contributing to the rise in respiratory infections observed in 2022.

Unlike other conditions, we did not observe an increase in hospitalisations for pneumococcal disease following ED presentation in 2022 when COVID‐19 restrictions were lifted. One possible reason for the stable proportion could be the substantial increase in other respiratory infections, such as RSV infection, which might have influenced the overall ARI hospitalisation patterns. This observation is supported by recent data indicating a resurgence of invasive pneumococcal disease (IPD) in children post‐COVID, as documented in a study across three NSW hospitals [35]. Additionally, it is possible that the new pneumococcal vaccination recommendations implemented in July 2020, which recommended a single dose of PCV13 at age 70 for older Australians without risk conditions, replacing the previous PPV23 at age 65, might have also contributed to our observations [36]. However, further studies are needed to confirm this hypothesis.

Although ARI‐like ED presentation rates were highest among children aged 0–4 years, hospitalisation rates for specific respiratory conditions were lower than in adults aged 65 years and above. The exception was RSV hospitalisations, which peaked in 2022. Previous research supports these findings, reporting a resurgence of RSV infection following the COVID‐19 pandemic in many countries, including Australia, particularly among children aged 2–5 years [37, 38]. Another study also noted a re‐emergence of RSV infection in Australian children after COVID‐19 public health measures were lifted [39]. This increased rates among young children may be partly due to the concept of “immunity debt,” where reduced exposure to common pathogens during COVID‐19 restrictions has led to decreased immune stimulation [25]. As a result, children who did not encounter these infections during the pandemic are now more susceptible as normal activities resume [25].

### 4.1 Strengths and Limitations

This study represents a population‐based analysis leveraging linked administrative data from a large and diverse Australian population, encompassing both highly urbanised and remote regional areas. By tracking trends in ARI‐like ED presentations over a 12‐year period, it provides a comprehensive examination of the burden of acute respiratory infections on the hospital system. Additionally, the study offers a detailed analysis of hospitalisation trends for specific respiratory conditions and subsequent mortality rates, effectively capturing the patient journey from the ED presentations to death. Study limitations include that the EDDC and APDC are administrative databases and therefore lack detailed clinical information to further define disease states. Another important limitation is the potential underestimation of hospitalisations for specific infections, particularly in adults, due to not everyone being tested. This can result in incomplete data on the prevalence and severity of these infections. For example, RSV hospitalisations may be categorised under nonspecific RSV codes, meaning that estimates based solely on diagnostic codes might not fully reflect the actual burden, especially among older adults [32]. Lastly, using 28‐day mortality rates following ARI‐like ED presentations as a measure does not specify the causes of death. This limitation arises because the infection may not have been the direct cause of death but could have accelerated it. Additionally, there may have been deaths that occurred coincidentally with the infection, making it challenging to determine the exact impact of the infection on mortality rates.

## 5 Conclusions

In summary, our study provides insights into the outcomes of patients with vaccine‐preventable and other acute respiratory infections over more than a decade. It highlights the critical importance of monitoring trends in these infections to better understand their epidemiology and assess the effectiveness of public health interventions, including vaccination programmes and other COVID‐19 control measures. These findings offer valuable insights that can guide policies aimed at improving vaccination coverage and managing the burden of these diseases.

To further enhance the impact of such surveillance, future research should focus on expanding data linkage efforts to include the vaccination status of admitted patients and other relevant variables at the individual level. This approach would enable more precise assessments of vaccine effectiveness and the long‐term impact of public health interventions, ultimately leading to more targeted and effective policy decisions.

## Author Contributions

**Fariha Binte Hossain:** writing – original draft, writing – review and editing, formal analysis, data curation, conceptualization, methodology, visualization. **David Muscatello:** conceptualization, supervision, writing – review and editing, methodology. **Sanjay Jayasinghe:** writing – review and editing, supervision, methodology. **Bette Liu:** conceptualization, writing – review and editing, supervision, methodology, investigation, validation.

## Conflicts of Interest

One of the authors, David Muscatello, reports grants from the National Health and Medical Research Council (NHMRC), Australia, during the conduct of the study. Personal fees are from PAHO, WHO, and NSW Ministry of Health, outside the submitted work. Other authors have nothing to disclose.

### Peer Review

The peer review history for this article is available at https://www.webofscience.com/api/gateway/wos/peer‐review/10.1111/irv.70015.

## Supporting information


**Table S1** Emergency department (ED) discharge diagnoses:ICD‐9‐AM, ICD‐10‐AM and SNOMED‐CT codes for acute respiratory infections (ARI) and related symptoms.
Table S2: ICD‐10‐AM codes for acute respiratory infection and their description.
Table S3: 28‐day mortality rate following ED presentations among those who were hospitalised, by respiratory infection categories and age group.

## Data Availability

The data that support the findings of this study are available on request. The data are not publicly available due to privacy or ethical restrictions.
