# Supplementary material for: Trends in Hospitalisations for Vaccine Preventable Respiratory Infections Following Emergency Department Presentations in New South Wales, Australia, 2012–2022
Source: Influenza Other Respir Viruses. 2024 Sep 26;18(10):e70015. doi: 10.1111/irv.70015 (PMC11427497; doi:10.1111/irv.70015)
Supplement: Supplementary file 1 — Table S1 Emergency department (ED) discharge diagnoses:ICD‐9‐AM, ICD‐10‐AM and SNOMED‐CT codes for acute respiratory infections (ARI) and related symptoms. Table S2: ICD‐10‐AM codes for acute respiratory infection and their description. Table S3: 28‐day mortality rate following ED presentations among those who were hospitalised, by respiratory infection categories and age group. [file IRV-18-e70015-s001.docx]

**Title of the study**

Trends in hospitalisations for vaccine preventable respiratory infections following emergency department presentation in NSW, 2012-2022.

**Supplementary table 1:** Emergency department (ED) discharge diagnoses: ICD-9-AM, ICD-10-AM, and SNOMED-CT codes for acute respiratory infections (ARI) and related symptoms

| Clinical codeset | ED diagnosis codes | Diagnosis description |
| --- | --- | --- |
| ICD-10-AM | J951  R04.2  J39  J39.2  J39.3  J39.9  J390  J392  J399  J98.9  J988  J988A  J988B  J989  R09.89  R070  R09.1  R091  J32  P28.40  P283  P2840  R06.1  R06.3  R06.4  R06.5  R06.8  R061  R064  R068  R09.0  R090  J80  J96.0  J96.09  J96.9  J96.99  J969  J9699  P22  P22.0  P22.9  R09.2  R092  J95.1  P28  P28.4  P28.83  P28.9  P284  R06  R06.7  R06.2  R062  J21  J21.0  J21.9  J219  B34.2  B97.2  U07.1  U07.2  U071  U072  R05  A37  A37.9  A379  A49.9  A499  R50  R50.0  R50.1  R50.8  R50.9  R509  R509A  R509B  R509C  B99  A68.1  A99  B96.88  B9688  B34.9  B349  B97.8  J10.0  J10.1  J100  J101  J11  J11.0  J11.1  J11.8  J111  J05.1  J051  J03.0  J04.1  J041  J20.2  J36  J20  J20.9  J209  J40  J05  J05.0  J050  J22  J02.8  J04  J04.0  J04.2  J040  J042  J06.0  J00  J39.0  J39.8  J98.8  J02  J02.0  J02.9  J020  J029  R07.0  J01  J01.0  J01.1  J01.9  J010  J011  J019  J03  J03.8  J03.9  J039  J06  J06.8  J06.9  J068  J069  B01.2  J12  J12.8  J12.9  J129  J13  J15  J15.7  J15.8  J15.9  J157  J159  J16  J16.0  J17.0  J18  J18.0  J18.1  J18.8  J18.9  J180  J181  J188  J188A  J189  A40.9  A41  A41.8  A41.9  A418  A419  R57.2 | Acute pulmonary insufficiency following thoracic surgery  Haemoptysis, haemorrhage from respiratory passages  Other diseases of upper respiratory tract  Other diseases of pharynx  Upper respiratory tract hypersensitivity reaction, site unspecified  Disease of upper respiratory tract, unspecified  Retropharyngeal and parapharyngeal abscess  Other diseases of pharynx  Disease of upper respiratory tract, unspecified  Respiratory disorder, unspecified  Other specified respiratory disorders  Other specified respiratory disorders  Other specified respiratory disorders  Respiratory disorder, unspecified  Other specified symptoms and signs involving the respiratory system  Pain in throat  Pleurisy  Pleurisy  Chronic sinusitis  Apnoea of newborn, unspecified  Primary sleep apnoea of newborn  Apnoea of newborn, unspecified  Stridor  Periodic breathing  Hyperventilation  Mouth breathing  Other and unspecified abnormalities of breathing  Stridor  Hyperventilation  Other and unspecified abnormalities of breathing  Asphyxia  Asphyxia  Acute respiratory distress syndrome  Acute respiratory failure  Acute respiratory failure, type unspecified  Respiratory failure, unspecified  Respiratory failure unspecified, type unspecified  Respiratory failure, unspecified  Respiratory failure unspecified, type unspecified  Respiratory distress of newborn  Respiratory distress syndrome of newborn  Respiratory distress of newborn, unspecified  Respiratory arrest  Respiratory arrest  Acute pulmonary insufficiency following thoracic surgery  Other respiratory conditions originating in the perinatal period  Other apnoea of newborn  Grunting in newborn  Respiratory condition of newborn, unspecified  Other apnoea of newborn  Cough  Sneezing  Wheezing  Wheezing  Acute bronchiolitis  Acute bronchiolitis due to respiratory syncytial virus  Acute bronchiolitis, unspecified  Acute bronchiolitis, unspecified  Coronavirus infection, unspecified site  Coronavirus as the cause of diseases classified to other chapters  Coronavirus disease 2019 [COVID-19], virus identified  Coronavirus disease 2019 [COVID-19], virus not identified  Coronavirus disease 2019 [COVID-19], virus identified  Coronavirus disease 2019 [COVID-19], virus not identified  Cough  Whooping cough  Whooping cough, unspecified  Whooping cough, unspecified  Bacterial infection, unspecified  Bacterial infection, unspecified  Fever of other and unknown origin  Fever with chills  Persistent fever  Other specified fever  Fever, unspecified  Fever, unspecified  Fever, unspecified  Fever, unspecified  Fever, unspecified  Other and unspecified infectious diseases  Tick-borne relapsing fever  Unspecified viral haemorrhagic fever  Other and unspecified bacterial agents as the cause of diseases classifi  Other and unspecified bacterial agents as the cause of diseases classifi  Viral infection, unspecified  Viral infection, unspecified  Other viral agents as the cause of diseases classified to other chapters  Influenza with pneumonia, other influenza virus identified  Influenza with other respiratory manifestations, other influenza virus i  Influenza with pneumonia, other influenza virus identified  Influenza with other respiratory manifestations, other influenza virus i  Influenza, virus not identified  Influenza with pneumonia, virus not identified  Influenza with other respiratory manifestations, virus not identified  Influenza with other manifestations, virus not identified  Influenza with other respiratory manifestations, virus not identified  Acute epiglottitis  Acute epiglottitis  Streptococcal tonsillitis  Acute tracheitis  Acute tracheitis  Acute bronchitis due to Streptococcus  Peritonsillar abscess  Acute bronchitis  Acute bronchitis, unspecified  Acute bronchitis, unspecified  Bronchitis, not specified as acute or chronic  Acute obstructive laryngitis [croup] and epiglottitis  Acute obstructive laryngitis [croup]  Acute obstructive laryngitis [croup]  Unspecified acute lower respiratory infection  Acute pharyngitis due to other specified organisms  Acute laryngitis and tracheitis  Acute laryngitis  Acute laryngotracheitis  Acute laryngitis  Acute laryngotracheitis  Acute laryngopharyngitis  Acute nasopharyngitis [common cold]  Retropharyngeal and parapharyngeal abscess  Other specified diseases of upper respiratory tract  Other specified respiratory disorders  Acute pharyngitis  Streptococcal pharyngitis  Acute pharyngitis, unspecified  Streptococcal pharyngitis  Acute pharyngitis, unspecified  Pain in throat  Acute sinusitis  Acute maxillary sinusitis  Acute frontal sinusitis  Acute sinusitis, unspecified  Acute maxillary sinusitis  Acute frontal sinusitis  Acute sinusitis, unspecified  Acute tonsillitis  Acute tonsillitis due to other specified organisms  Acute tonsillitis, unspecified  Acute tonsillitis, unspecified  Acute upper respiratory infections of multiple and unspecified sites  Other acute upper respiratory infections of multiple sites  Acute upper respiratory infection, unspecified  Other acute upper respiratory infections of multiple sites  Acute upper respiratory infection, unspecified  Varicella pneumonia  Viral pneumonia, not elsewhere classified  Other viral pneumonia  Viral pneumonia, unspecified  Viral pneumonia, unspecified  Pneumonia due to Streptococcus pneumoniae  Bacterial pneumonia, not elsewhere classified  Pneumonia due to Mycoplasma pneumoniae  Other bacterial pneumonia  Bacterial pneumonia, unspecified  Pneumonia due to Mycoplasma pneumoniae  Bacterial pneumonia, unspecified  Pneumonia due to other infectious organisms, not elsewhere classified  Chlamydial pneumonia  Pneumonia in bacterial diseases classified elsewhere  Pneumonia, organism unspecified  Bronchopneumonia, unspecified  Lobar pneumonia, unspecified  Other pneumonia, organism unspecified  Pneumonia, unspecified  Bronchopneumonia, unspecified  Lobar pneumonia, unspecified  Other pneumonia, organism unspecified  Other pneumonia, unspecified organism  Pneumonia, unspecified  Streptococcal sepsis, unspecified  Other and unspecified sepsis  Sepsis due to other specified organism  Sepsis, unspecified  Sepsis due to other specified organism  Sepsis, unspecified  Septic shock |
| ICD-9-AM | 51882  518.81  4661  4660  5185  511.9  5119  511  518.82  786  786.01  786.02  786.9  78601  78602  7869  518.81  769  799.1  7991  7862  518.5  786.8  7868  519.9  5198  5199  786.09  78609  799  7990  786.1  466.1  786.2  33  33.9  339  780.6  7806  136.9  1369  41.9  419  79.9  79.99  799  790.7  790.8  7908  487  487  487.1  487.4  487.8  4870  4871  464.3  46430  34  340  464.1  46410  475  466  466  490  464  464.2  464.2  4640  46420  460  519.8  462  461  461.1  461.8  461.9  4610  4611  4618  4619  463  465.8  465.9  4658  4659  480.9  4809  481  482.9  4829  483  484.8  485  486  38  38.9  389  7907 | Other pulmonary insufficiency, not elsewhere classified  Acute respiratory failure  Acute bronchiolitis  Acute bronchitis  Pulmonary Insufficiency  Unspecified pleural effusion  Unspecified pleural effusion  Pleurisy without mention of effusion or current tuberculosis  Other pulmonary insufficiency, not elsewhere classified  Respiratory abnormality, unspecified  Hyperventilation  Orthopnea  Other symptoms involving respiratory system and chest  Hyperventilation  Orthopnea  Other symptoms involving respiratory system and chest  Acute respiratory failure  Respiratory distress syndrome in newborn  Respiratory arrest  Respiratory arrest  Cough  Pulmonary Insufficiency  Abnormal chesh sounds, hiccough  Hiccough  Unspecified disease of respiratory system  Other diseases of respiratory system, not elsewhere classified  Unspecified disease of respiratory system  Other respiratory abnormalities  Other respiratory abnormalities  Unspecified viral and chlamydial infections  Unspecified viral and chlamydial infections  Stridor  acute bronchiolitis  Cough  Whooping cough due to bordetella pertussis [B. pertussis]  Whooping cough, unspecified organism  Whooping cough, unspecified organism  Fever  Fever  Unspecified infectious and parasitic diseases  Unspecified infectious and parasitic diseases  Bacterial infection, unspecified, in conditions classified elsewhere and  Bacterial infection, unspecified, in conditions classified elsewhere and  Unspecified viral and chlamydial infections  Unspecified viral infection  Unspecified viral and chlamydial infections  Bacteremia  Viremia, unspecified  Viremia, unspecified  Influenza with pneumonia  Influenza with pneumonia  Influenza with other respiratory manifestations  Influenza unspecified  Influenza with other manifestations  Influenza with pneumonia  Influenza with other respiratory manifestations  Acute epiglottitis without mention of obstruction  Acute epiglottitis without mention of obstruction  Streptococcal sore throat  Streptococcal sore throat  Acute tracheitis without mention of obstruction  Acute tracheitis without mention of obstruction  Peritonsillar abscess  Acute bronchitis  Acute bronchitis  Bronchitis, not specified as acute or chronic  Acute laryngitis without mention of obstruction  Acute laryngotracheitis without mention of obstruction  Acute laryngotracheitis without mention of obstruction  Acute laryngitis without mention of obstruction  Acute laryngotracheitis without mention of obstruction  Acute nasopharyngitis [common cold]  Other diseases of respiratory system, not elsewhere classified  Acute pharyngitis  Acute maxillary sinusitis  Acute frontal sinusitis  Other acute sinusitis  Acute sinusitis, unspecified  Acute maxillary sinusitis  Acute frontal sinusitis  Other acute sinusitis  Acute sinusitis, unspecified  Acute tonsillitis  Acute upper respiratory infections of other multiple sites  Acute upper respiratory infections of unspecified site  Acute upper respiratory infections of other multiple sites  Acute upper respiratory infections of unspecified site  Viral pneumonia, unspecified  Viral pneumonia, unspecified  Pneumococcal pneumonia [Streptococcus pneumoniae pneumonia]  Bacterial pneumonia, unspecified  Bacterial pneumonia, unspecified  Pneumonia due to mycoplasma pneumoniae  Pneumonia in other infectious diseases classified elsewhere  Bronchopneumonia, organism unspecified  Pneumonia, organism unspecified  Streptococcal septicemia  Unspecified septicemia  Unspecified septicemia  Bacteremia |
| SNOMED-CT | 1023001  110289004  111282000  13094009  15993004  161941007  162375000  162481009  162899009  181893002  20573003  206324006  206996006  207050008  207053005  207107001  225589000  230145002  248548009  248552009  248556007  248575002  248581005  248584002  248585001  271823003  271877004  276544005  281003006  289105003  29596007  307487006  313246000  31515003  373909009  38593000  386614005  386813002  389086002  39549008  399164003  399322006  405272003  409623005  416945002  47525004  60118008  60845006  62744007  63214000  68978004  713013008  75483001  77329001  90480005  95617006  162711000  162892000  207055003  207552005  207553000  207554006  233765002  248583008  271825005  274282003  276259003  34681009  371044001  373895009  397767007  39871006  409622000  46775006  65710008  67782005  67905004  709110009  87317003  95634003  161923004  161924005  161925006  289116005  248574003  162890008  17077006  17216000  20112008  207051007  207052000  207057006  207059009  248549001  248565000  25209001  263653002  267036007  271824009  276531000  297216006  301820005  308149007  34560001  386616007  39950000  422177004  55442000  57769004  59265000  67158008  72365000  73322006  80954004  95616002  85617008  110291007  111489007  233712009  247813005  248578000  248626009  25423001  276708003  289102000  301245004  301282008  301283003  301285005  361208003  366138001  392562007  398234006  57000008  65958008  90091006  90661000  95430002  106012007  129893005  162951005  17849001  206998007  240310001  248573009  271375003  276545006  276725001  301273002  301826004  309155007  40296006  42908004  58596002  62085005  70073005  724229002  72863001  7550008  79451004  95611007  162367006  164274003  206999004  207064008  24612001  275280004  301287002  70407001  76067001  162894004  18197001  207058001  272040008  301703002  31572008  56018004  68095009  9763007  59903001  13089009  15199004  195737004  233602006  233603001  4120002  52409006  5505005  718004  195739001  398447004  186747009  27619001  713084008  840533007  840539006  19282004  248594007  68154008  111962006  11833005  135883003  161927003  161929000  161932002  161933007  161947006  162480005  20670007  207066005  207067001  247410004  248592006  248593001  263731006  272039006  276314008  284523002  28743005  301236000  315246003  409596002  417850002  43025008  46789001  49727002  59994004  62427007  62548007  62618004  62731002  63000007  7142008  17986004  289965001  249596001  300959008  161422003  26183002  26484003  271567008  27836007  408682005  5247005  58217006  77116006  82670009  255320000  371582002  371583007  102496004  103001002  164285001  164288004  164299002  164306004  164307008  164308003  164309006  164311002  164312009  164314005  206758001  206760004  206763002  248427009  248430002  248431003  248434006  248435007  248445009  248449003  271749004  271750004  271751000  271753002  271897009  274234009  274640006  285717004  304213008  365977007  373904004  373906002  386661006  38880002  409702008  416113008  420079008  421154002  42136008  426000000  50177009  70226007  722892007  7520000  77957000  9619006  40733004  63171007  87628006  409089005  111794008  115648009  164287009  168197002  193198003  240523007  250465006  264569006  271754008  28456004  312420009  63459005  105636004  161424002  207295008  2528003  312085005  312130009  34014006  409630004  409631000  409798003  445939008  73198007  275719008  95891005  118089005  120753009  121006005  122349003  194946005  195878008  195920000  195923003  195924009  195929004  24662006  2731000  309789002  309806000  315142009  315642008  359829002  359833009  407477006  407478001  407479009  407480007  41269000  420362005  42964004  43692000  442438000  442696006  46171006  55014007  55604004  6142004  61700007  63039003  78431007  84037004  408687004  109362006  11296007  21060003  222008  232432003  232433008  248554005  248555006  29608009  47125007  49908003  56937005  58576005  70976000  73342002  79877004  80384002  15033003  164255006  18099001  186357007  195658003  195671000  195673002  195677001  195707008  195721005  233598009  26650005  33261009  41582007  43878008  45629003  50211006  59707005  62994001  64369009  75498004  84889008  95886009  195712009  195728004  57089007  10509002  16146001  185086009  195714005  195717003  195720006  195722003  195725001  195726000  195727009  233599001  233600003  233601004  27475006  275499005  312371005  312400008  32398004  35301006  36426008  5875001  65878001  79479005  80257001  89549007  275495004  71186008  135882008  195647007  195742007  275497007  275498002  312119006  312133006  312134000  419502003  448739000  50417007  10809006  195680000  195681001  195682008  195683003  195684009  195686006  232426008  232428009  276443001  301824001  308232009  35377009  37948003  408669002  45913009  55130001  55355000  64375000  6655004  71255007  72204002  85083002  85915003  82272006  102618009  116277006  126665005  195663004  69061003  707351006  128601007  195708003  205237003  300281007  308130008  408418009  68372009  66011008  90389005  102617004  126664009  1532007  162388002  162397003  195655000  195656004  195657008  195659006  195660001  195662009  195709006  195836002  267102003  275488008  312422001  363746003  39271004  405737000  51476001  58031004  59471009  78430008  78911000  312117008  15805002  195788001  232391008  30239003  36971009  371127003  40055000  5028002  61711004  67832005  68272006  77919000  82297005  91038008  102453009  10351008  14465002  17741008  195666007  195667003  195668008  195669000  195670004  195676005  195804009  232418000  240444009  27878001  281795003  302911003  51209006  90176007  195747001  232343007  275496003  281794004  312118003  54150009  54398005  61958003  78337007  195889001  195902009  195911009  26726000  312403005  32286006  3487004  46207001  61884008  64703005  85469005  385093006  195900001  408679000  408680002  415125002  416916004  425464007  76090006  16810008  195881003  41207000  64917006  111900000  123587001  123588006  123590007  123591006  123592004  161525004  191727003  195886008  195908008  195909000  22754005  233604007  233606009  233607000  233609002  233613009  233621003  266350000  278516003  300999006  301000005  301001009  301002002  301003007  301004001  308906005  312342009  34020007  35037009  396284006  396285007  396286008  407671000  409664000  409665004  409804001  41381004  420544002  420787001  421671002  46970008  51530003  53084003  55679008  57702005  64479007  66429007  699014000  70036007  75570004  81164001  95436008  59475000  233785003  233795005  4089001  10001005  105592009  194394004  207031008  234173007  234174001  238150007  361206004  443980004  447899008  447931005  448417001  449082003  53869006  76571007  79587009  91302008 | Apnea (finding)  Obligatory mouth breathing (finding)  Acute respiratory insufficiency (disorder)  Apnea in newborn (finding)  Alveolar hypoventilation (finding)  Dyspnea at rest (finding)  Snoring symptoms (finding)  Breathing aggravates symptom (finding)  On examination - hyperventilating  Excessively deep breathing (finding)  Respiratory pattern impairment  Neonatal acrocyanosis (finding)  [D]Choking sensation  [D]Hyperventilation  [D]Apnoea  [D]Breath-holding spell  Chokes when swallowing (finding)  Difficulty breathing (finding)  Nocturnal dyspnea (finding)  Winded (finding)  Choking during respiration (finding)  Groaning respiration (finding)  Panting  Catching breath (finding)  Irregular breathing (finding)  Tachypneic  Suffocating (finding)  Apnea of prematurity (disorder)  Choking attacks (finding)  Difficulty controlling breathing (finding)  Hypercapnia (disorder)  Biphasic stridor (finding)  [D]Sleep apnoea syndrome  Hypoventilation (finding)  Sense of suffocation  Perinatal cyanotic attacks (finding)  Oligopnoea  Breathing abnormal  Decreased oxygen supply  Chronic hypoventilation (finding)  Acidotic hyperventilation (finding)  Air hunger (finding)  Primary alveolar hypoventilation  Pulmonary insufficiency  Recurrent apnea  Coarse respiratory crackles (finding)  Acute respiratory alkalosis (disorder)  Short of breath on exertion  Breathlessness lying flat  Vagal apnea (finding)  Hyperventilation (finding)  Breath holding spell (finding)  Breathing painful (finding)  Breathing orally  Interrupted breathing (finding)  Neonatal cyanosis (disorder)  On examination - collapse - respiratory arrest  On examination - respiratory distress  [D]Respiratory distress  [D]Respiratory failure  [D]Cardiorespiratory failure  [D]Respiratory arrest  Respiratory failure without hypercapnia (disorder)  Stops breathing (finding)  Respiratory distress (finding)  On examination - respiratory arrest  Respiratory arrest preceding cardiac arrest (disorder)  Perinatal respiratory distress (disorder)  Transient respiratory distress with sepsis (disorder)  Acute respiratory distress  Acute respiratory failure requiring reintubation (disorder)  Chronic respiratory failure (disorder)  Respiratory failure (disorder)  Respiratory distress syndrome in neonate  Acute respiratory failure (disorder)  Acute respiratory distress syndrome (disorder)  Acute on chronic respiratory failure  Acute hypercapnic respiratory failure  Respiratory arrest (disorder)  Neonatal respiratory arrest (disorder)  Productive cough -clear sputum (finding)  Productive cough -green sputum (finding)  Productive cough-yellow sputum (finding)  Difficulty coughing (finding)  Croupy breathing (finding)  On examination - dyspnoea  Abnormal respiratory rate (finding)  Dyspnea, class I (finding)  Dyspnea leaning over (finding)  [D]Orthopnoea  [D]Tachypnoea  [D]Shortness of breath  [D]Dyspnoea  Labored breathing (finding)  Respiratory effort (observable entity)  Inspiratory dyspnea (finding)  Abdominal respiratory movements (observable entity)  Dyspnea (finding)  Respiration intermittent (finding)  Mild transient tachypnoea of newborn  Increasing breathlessness (finding)  Finding of respiratory effort (finding)  [D]Breathlessness  Expiratory dyspnea (finding)  Shallow breathing  Dyspnoea, class 3  Dyspnea with AIDS (acquired immunodeficiency syndrome)  Paroxysmal nocturnal dyspnea (finding)  Dyspnea after eating (finding)  Paroxysmal dyspnea (finding)  Tracheal tug (finding)  Dyspnea, class 2  Dyspnoea, class 4  Decreased respiratory function (finding)  Neonatal apneic attack (finding)  Abnormal respiratory rhythm (finding)  Chronic mouth breathing (finding)  Breathing-related sleep disorder (disorder)  Fluid overload pulmonary edema (disorder)  Phobia of choking  Sobbing respiration (finding)  Yawning (finding)  Sighing respiration (finding)  Infant slow to establish respiration (finding)  Unable to control breathing (finding)  Difficulty in coughing up sputum (finding)  Finding of respiration (finding)  Finding of rate of respiration (finding)  Finding of sound of breathing (finding)  Primary sleep apnea of newborn (disorder)  Finding of respiratory sounds  Risk for sudden infant death syndrome  Breathing system problem (event)  Traumatic apnea (finding)  Singultus/hiccough  Breath holding with temper (finding)  Diaphragmatic tonic spasm (finding)  Respiratory tract paralysis (disorder)  Hypoxia, asphyxia AND/OR other respiratory condition of foetus AND/OR ne  Respiratory alteration (finding)  On examination - bronchial breathing  Foetus and newborn respiratory conditions  [D]Mouth breathing  Grunting baby (finding)  Noisy respiration (finding)  Snuffles in newborn (finding)  Obstructive apnea of newborn (disorder)  Recurrent apnea of newborn (finding)  Abnormal breath sounds (finding)  Intermittent stridor (finding)  Cheyne Stokes respiration  Meningitic respiration (finding)  Primary atelectasis, in perinatal period (disorder)  Inspiratory stridor (finding)  Grunting respiration (finding)  Perinatal apneic spells (disorder)  Apnea of infancy  Observation of snoring  Wet lung syndrome in newborn  Bronchial breathing (finding)  Neonatal hypoventilation (finding)  Sneezing symptom  On examination - stridor present  [D]Sneezing  [D]Stridor  Rhonchus  Sniffles (finding)  Expiratory stridor (finding)  Stridor (finding)  Observation of sneezing  On examination - expiratory wheeze  Asthmatoid wheeze (finding)  [D]Wheezing  Wheezing symptom (finding)  Expiratory polyphonic wheeze (finding)  Inspiratory wheezing (finding)  Wheezing (finding)  Wheezing stridor (finding)  Expiratory wheezing (finding)  Acute obliterating bronchiolitis (disorder)  Adenoviral bronchiolitis (disorder)  Acute bronchiolitis with bronchospasm (disorder)  Acute exudative bronchiolitis (disorder)  Acute viral bronchiolitis (disorder)  Acute bronchiolitis caused by adenovirus  Bronchiolitis (disorder)  Bronchiolitis exudativa (disorder)  Acute capillary bronchiolitis  Acute bronchiolitis with obstruction (disorder)  Acute bronchiolitis caused by respiratory syncytial virus  Severe acute respiratory syndrome  Coronavirus infection (disorder)  Disease caused by Coronaviridae  Pneumonia caused by Human coronavirus  Severe acute respiratory syndrome coronavirus 2 (organism)  Disease caused by SARS-CoV-2  Cough on exercise (finding)  Tracheal esophageal fistula cough (finding)  Chronic cough (finding)  Postural cough (finding)  Non-productive cough  Cough with fever (finding)  Night cough present (situation)  Chesty cough (finding)  Morning cough (finding)  Evening cough (finding)  Nocturnal cough / wheeze (finding)  Cough aggravates symptom (finding)  Brassy cough (finding)  [D]Cough  [D]Cough syncope  Painful cough (finding)  Character of cough (observable entity)  Cough when swallowing (finding)  Coughing (observable entity)  Complaining of cough  Coughing ineffective (finding)  Persistent cough (finding)  Bronchial cough  Effective cough (finding)  Unexplained cough (finding)  Non-productive cough (finding)  Respiratory tract congestion and cough (disorder)  Paroxysmal cough (finding)  Cough after eating (finding)  Observation of cough  Hacking cough (finding)  Bovine cough (finding)  Nocturnal cough (finding)  Early morning cough (finding)  Increasing frequency of cough (finding)  Spasmodic cough (finding)  Cough at rest (finding)  Barking cough (finding)  Croupy cough (finding)  Cough impulse in inguinal canal (finding)  Allergic cough (finding)  History of pertussis  Bordetella parapertussis (organism)  Bordetellosis (disorder)  Whooping cough-like syndrome (disorder)  Pertussis (disorder)  Healthcare associated pertussis (disorder)  Bordetella pertussis (organism)  Infection caused by Bordetella bronchiseptica  Infection caused by Bordetella parapertussis  Whooping cough caused by organism other than Bordetella pertussis  Infection - suppurative (disorder)  Disease caused by Gram-positive bacteria  Disease caused by Gram-negative bacteria  Spiking temperature  Feels feverish  On examination - fever - general  On examination - pyrexia of unknown origin  On examination - level of fever  On examination - character of fever  On examination - fever - acute rise  On examination - fever - gradual rise  On examination - fever - continuous  On examination - fever - remittent  On examination - fever - intermittent  On examination - fever - irregular  [D]Pyrexia of unknown origin  [D]Chills with fever  [D]Persistent fever  Fever symptoms (finding)  Character of fever  Phase of fever (observable entity)  Falling phase of fever (finding)  Prolonged fever (finding)  Fever, diurnal variation (finding)  Central fever (finding)  Acute rise of fever (finding)  Gradual rise of fever (finding)  Continuous fever (finding)  Irregular fever (finding)  On examination - fever  [D]Pyrexia (situation)  Fever with rigors  High temperature (physical force)  Low grade fever  Finding of pattern of fever  Feels hot/feverish  Fever with sweating  Fever  Rigors  Hyperpyrexia (finding)  Febrile illness (fever)  Recurrent fever  Fever associated with acquired immunodeficiency syndrome  Swinging fever (finding)  Fever greater than 38 Celsius  High body temperature  Heat pyrexia (disorder)  Fever due to infection (finding)  Pyrexia of unknown origin (finding)  Intermittent fever (finding)  Aseptic fever (finding)  Infectious disease (disorder)  Acute infectious disease (disorder)  Bacterial infectious disease (disorder)  Neutropaenic fever  Masked infection (disorder)  Persistent infection (disorder)  On examination - fever examination - no abnormality detected (finding)  Sample bacteria cultured (finding)  Suprainfection  Viral hemorrhagic fever (disorder)  Finding of growth of bacteria  Secondary infection (disorder)  Rapid fall of fever (finding)  Subclinical infection (disorder)  Infection of oral cavity, teeth and salivary gland (disorder)  Clinical infection (disorder)  Disease of possible viral origin (disorder)  History of viral illness (situation)  [D]Unspecified viraemia  Viremia (finding)  Infection - non-suppurative (disorder)  Viral infection by site (disorder)  Disease due to virus  Asymptomatic viremia (finding)  Acute viral disease (disorder)  Infection caused by resistant virus  Nonspecific syndrome suggestive of viral illness  Bacterial pleurisy (disorder)  Sputum evidence of infection (finding)  Influenza like illness  Influenza A virus and Influenza B virus antigen assay (procedure)  Influenza A virus antibody  Antigen of Influenza A virus (substance)  Influenza A virus antigen assay (procedure)  Acute myocarditis - influenzal (disorder)  Pneumonia and influenza (disorder)  Influenza with pneumonia, influenza virus identified (disorder)  Influenza with laryngitis (disorder)  Influenza with pharyngitis (disorder)  Influenza with gastrointestinal tract involvement (disorder)  Influenza caused by Influenza B virus (disorder)  Serologic test for Influenza A virus (procedure)  Encephalitis caused by influenza  Encephalitis caused by influenza-virus identified (disorder)  Influenza A antibody level (procedure)  Influenza-like symptoms (finding)  Avian influenzavirus  Swine influenzavirus  Genus Alphainfluenzavirus  Genus Betainfluenzavirus (organism)  Influenza virus A  Influenza B virus  Influenzal bronchopneumonia (disorder)  Influenzavirus type A, avian, H1N1 strain  Primary influenza virus pneumonia  Influenzal acute upper respiratory infection (disorder)  Influenza caused by Influenza A virus (disorder)  Influenza caused by Influenza A virus subtype H1N1  Influenza due to Influenza virus, type A, porcine (disorder)  Orthomyxoviridae  Bird flu  Influenza (disorder)  Influenza with non-respiratory manifestation (disorder)  Influenza with respiratory manifestation other than pneumonia (disorder)  Influenza due to Influenza virus, type A, human (disorder)  Swine influenza (disorder)  Healthcare associated influenza disease  Cellulitis of parapharyngeal space (disorder)  Stenosis of trachea (disorder)  Acute bacterial epiglottitis (disorder)  Acute epiglottitis with obstruction (disorder)  Pediatric acute epiglottitis and supraglottitis (disorder)  Adult acute epiglottitis and supraglottitis (disorder)  Partially obstructed airway (disorder)  Total airway obstruction  Acute epiglottitis (disorder)  Disorder of trachea  Acute epiglottitis without obstruction (disorder)  Tracheal abscess  Haemophilus influenzae epiglottitis (disorder)  Viral epiglottitis (disorder)  Obstruction of trachea (disorder)  Bronchial stenosis  Epiglottitis (disorder)  Peritonsillar abscess (disorder)  On examination - tonsils - quinsy present  Retropharyngeal abscess (disorder)  Streptococcal sore throat with scarlatina (disorder)  Acute bacterial pharyngitis (disorder)  Acute bacterial tonsillitis (disorder)  Acute staphylococcal tonsillitis (disorder)  RAT - recurrent acute tonsillitis  Tracheopharyngitis (disorder)  Acute bronchitis caused by Haemophilus influenzae (disorder)  Acute bacterial bronchitis (disorder)  Acute tracheitis (disorder)  Abscess of tonsil (disorder)  Streptococcal tonsillitis (disorder)  Streptococcal sore throat (disorder)  Abscess of nasopharynx (disorder)  Catarrhal tracheitis (disorder)  Abscess of pharynx (disorder)  Tracheitis (disorder)  Acute tracheitis without obstruction (disorder)  Acute bacterial sinusitis (disorder)  Abscess of parapharyngeal space  Mycoplasmal tracheobronchitis (disorder)  Acute bronchitis and/or bronchiolitis (disorder)  Acute bronchitis caused by rhinovirus (disorder)  Respiratory syncytial virus bronchiolitis (disorder)  Acute bronchitis (disorder)  Viral bronchitis (disorder)  Chronic obstructive bronchitis  Acute pseudomembranous bronchitis  Acute purulent bronchitis (disorder)  Acute streptococcal bronchitis (disorder)  Acute Moraxella catarrhalis bronchitis (disorder)  Acute bronchitis caused by coxsackievirus  Acute parainfluenza virus bronchitis (disorder)  Acute respiratory syncytial virus bronchitis (disorder)  Acute mycoplasmal bronchitis (disorder)  Acute chlamydial bronchitis (disorder)  Acute viral bronchitis (disorder)  Parainfluenza virus bronchitis (disorder)  Acute wheezy bronchitis (disorder)  Acute infective bronchitis (disorder)  Acute infective tracheobronchitis (disorder)  Bronchitis (disorder)  Acute tracheobronchitis (disorder)  Subacute bronchitis (disorder)  Acute bronchitis with obstruction (disorder)  Septic bronchitis (disorder)  Respiratory syncytial virus bronchitis (disorder)  Acute bronchitis with bronchospasm (disorder)  Catarrhal bronchitis (disorder)  Acute fibrinous laryngotracheobronchitis (disorder)  Croup (disorder)  Feverish cold (finding)  Acute respiratory infection  Acute lower respiratory tract infection (disorder)  Infection of lower respiratory tract and mediastinum (disorder)  Respiratory tract infection (disorder)  Bacterial lower respiratory infection (disorder)  Viral respiratory infection (disorder)  Viral lower respiratory infection (disorder)  Chest infection (disorder)  Recurrent lower respiratory tract infection  Lower respiratory tract infection (disorder)  Parainfluenza virus laryngotracheitis (disorder)  Acute edematous laryngitis (disorder)  Acute ulcerative laryngitis (disorder)  Acute catarrhal laryngitis (disorder)  Acute phlegmonous laryngitis (disorder)  Acute laryngitis caused by Haemophilus influenzae (disorder)  Acute suppurative laryngitis (disorder)  Acute simple laryngitis (disorder)  Acute membranous laryngitis (disorder)  Acute laryngitis and/or tracheitis (disorder)  Acute viral laryngotracheitis (disorder)  Laryngotracheomalacia (disorder)  Parainfluenza virus laryngotracheobronchitis (disorder)  Acute laryngotracheitis without obstruction (disorder)  Acute laryngitis with obstruction  Laryngitis (disorder)  Laryngotracheitis (disorder)  Acute laryngopharyngitis (disorder)  Acute laryngotracheitis (disorder)  Acute laryngitis (disorder)  Adenoviral laryngotracheobronchitis (disorder)  Respiratory syncytial virus laryngotracheobronchitis (disorder)  Streptococcal laryngitis (disorder)  Laryngotracheobronchitis (disorder)  Cold  Pharyngeal dryness (finding)  Pulmonary aspiration of fluid (finding)  Oropharyngeal mucositis (disorder)  Allergic pharyngitis (disorder)  Extrinsic obstruction of trachea (disorder)  Respiratory infection, institutional  Infectious disease of lung (disorder)  Recurrent upper respiratory tract infection (disorder)  Pneumonitis (disorder)  Pharynx problem (finding)  Recurrent chest infection (disorder)  Upper airway resistance syndrome  Upper respiratory tract obstruction (disorder)  Viral tracheitis (disorder)  Rhinovirus  Congestion of pharynx  Exudative pharyngitis (disorder)  Viral pharyngitis (disorder)  Has a sore throat (situation)  Throat soreness  Acute gangrenous pharyngitis (disorder)  Acute phlegmonous pharyngitis (disorder)  Acute ulcerative pharyngitis (disorder)  Acute pneumococcal pharyngitis (disorder)  Acute staphylococcal pharyngitis (disorder)  Acute viral pharyngitis (disorder)  Pharyngolaryngitis (disorder)  Pharynx or nasopharynx abscess (disorder)  Sore throat symptom (finding)  Sore throat - chronic (finding)  Infective pharyngitis (disorder)  Acute pharyngitis (disorder)  Ulcerative pharyngitis (disorder)  Pharyngitis  Nasopharyngitis (disorder)  Suppurative pharyngitis (disorder)  Phlegmonous pharyngitis (disorder)  Adenoviral pharyngitis (disorder)  Parainfluenza virus pharyngitis (disorder)  Bacterial respiratory infection (disorder)  Acute inflammation of sinus  Recurrent sinusitis (disorder)  Recurrent acute sinusitis (disorder)  Acute abscess of sphenoidal sinus (disorder)  Sinusitis (disorder)  Obstructive sinusitis  Chronic infection of sinus  Acute pansinusitis (disorder)  Acute abscess of ethmoidal sinus (disorder)  Acute ethmoiditis  Acute maxillary sinusitis (disorder)  Acute sphenoidal sinusitis (disorder)  Congestion of nasal sinus (disorder)  Acute frontal sinusitis (disorder)  Acute peritonsillitis  Suppurative tonsillitis (disorder)  Ulcerative tonsillitis (disorder)  Acute tonsillitis (disorder)  Acute erythematous tonsillitis (disorder)  Acute follicular tonsillitis (disorder)  Acute ulcerative tonsillitis (disorder)  Acute catarrhal tonsillitis (disorder)  Acute gangrenous tonsillitis (disorder)  Acute viral tonsillitis (disorder)  Lingular tonsillitis (disorder)  Acute infection of tonsillar remnant (disorder)  Fusobacterial necrotizing tonsillitis (disorder)  Follicular tonsillitis (disorder)  Inflamed tonsils (finding)  Acute lingual tonsillitis (disorder)  Viral tonsillitis (disorder)  Tonsillitis (disorder)  Chest cold (disorder)  Acute irritant rhinitis (disorder)  Nasal infection (disorder)  Viral upper respiratory tract infection (disorder)  Bacterial upper respiratory infection (disorder)  Upper respiratory tract infection  Acute upper respiratory tract infection  Adenoviral respiratory disease (disorder)  Acute upper respiratory infection of multiple sites (disorder)  Legionnaires' disease  Anthrax pneumonia (disorder)  Chickenpox pneumonia (disorder)  Legionellosis  Legionnaire disease  Q fever pneumonia  Pulmonary candidiasis  Pneumonitis caused by acquired toxoplasmosis (disorder)  Achromobacter pneumonia (disorder)  Terminal bronchopneumonia (disorder)  Hypostatic pneumonia (disorder)  CAP - community acquired pneumonia  Pneumonia due to measles (disorder)  Healthcare associated pneumonia  Healthcare associated bacterial pneumonia (disorder)  Pneumocystosis jirovecii pneumonia (disorder)  Lipoid pneumonitis (disorder)  HAP - hospital acquired pneumonia  Tatlockia micdadei  Acquired immunodeficiency syndrome (AIDS) with viral pneumonia  Pneumonia caused by respiratory syncytial virus (disorder)  Adenoviral pneumonia (disorder)  Parainfluenza pneumonia  Pneumonia with aspergillosis  Acute bronchopneumonia (disorder)  Confluent bronchopneumonia with abscess formation (disorder)  Focal pneumonia (disorder)  Confluent pneumonia (disorder)  Acute mucous pneumonia (disorder)  History of pneumonia (situation)  Post measles pneumonia (disorder)  Group B streptococcal pneumonia (disorder)  Actinomycotic pneumonia (disorder)  Nocardial pneumonia (disorder)  Staphylococcal pneumonia (disorder)  Pneumonia (disorder)  Atypical pneumonia (disorder)  Pneumococcal pneumonia (disorder)  Pneumonia caused by Chlamydiaceae  Fungal pneumonia (disorder)  Rickettsial pneumonia (disorder)  Pneumococcal lobar pneumonia (disorder)  Lobar pneumonia (disorder)  Basal pneumonia (disorder)  Lobar pneumonia left lower lobe  Lobar pneumonia right lower lobe  Lobar pneumonia left upper lobe  Lobar pneumonia right middle lobe  Lobar pneumonia right upper lobe  Secondary bacterial pneumonia (disorder)  Infective pneumonia (disorder)  Streptococcal pneumonia  Primary atypical interstitial pneumonia (disorder)  Segmental pneumonia  Lobular pneumonia  Bilateral bronchopneumonia (disorder)  Bilateral pneumonia  Pneumonia caused by anaerobic bacteria  Pneumonia caused by aerobic bacteria  Infection caused by ESBL Klebsiella pneumoniae  Pseudomonal pneumonia  Bacterial pneumonia with acquired immunodeficiency syndrome  Pneumococcal pneumonia with acquired immunodeficiency syndrome  Pneumonia with acquired immunodeficiency syndrome (disorder)  Mycoplasma pneumoniae pneumonia  Pneumonia caused by Escherichia coli  Bacterial pneumonia (disorder)  Peribronchial pneumonia (disorder)  Unresolved pneumonia (disorder)  Pneumonia caused by Klebsiella pneumoniae (disorder)  Unresolved lobar pneumonia (disorder)  Recurrent pneumonia  Haemophilus influenzae pneumonia (disorder)  Viral pneumonia (disorder)  Ornithosis with pneumonia (disorder)  Lung consolidation (disorder)  Pneumonia with pertussis  Infection of trachea  Infection of bronchus  Meningococcemia (finding)  Bacterial sepsis (disorder)  Septicemia (disorder)  Severe sepsis with acute organ dysfunction caused by Gram-positive bacte  [D]Septic shock  Hyperdynamic septic shock (disorder)  Hypodynamic septic shock (disorder)  Sepsis syndrome (disorder)  Gram-positive septic shock (disorder)  Neutropenic sepsis (disorder)  Sepsis caused by Escherichia coli (disorder)  Clinical sepsis  Sepsis caused by Staphylococcus aureus (disorder)  Severe sepsis with acute organ dysfunction caused by Gram-negative bacte  Gram-negative septicemia  Septicemic shock  Septicemia caused by Bacteroides (disorder)  Sepsis (disorder) |

**Supplementary table 2:** ICD-10-AM codes for acute respiratory infection and their description

| **ARI (J00-J22, U071, U072, B34.2, B97.2, B97.4)** | | **All-cause pneumonia (J10.0, J11.0, J12-J18)** | | **Influenza (J09-J11)** | | **RSV infection (J12.1, J20.5, J21.0, B97.4)** | | **COVID-19 (U071, U072, B34.2, B97.2)** | | **Pneumococcal disease (J13, J18.1)** | |
| --- | --- | --- | --- | --- | --- | --- | --- | --- | --- | --- | --- |
| Code | Description | Code | Description | Code | Description | Code | Description | Code | Description | Code | Description |
| J00 | Acute nasopharyngitis [common cold] | J10.0 | Influenza with pneumonia, other influenza virus identified | J09 | Influenza due to identified zoonotic or pandemic influenza virus | J12.1 | Respiratory syncytial virus pneumonia | U071 | COVID- 19 virus identified | J13 | Pneumonia due to Streptococcus pneumoniae |
| J01 | Acute sinusitis | J11.0 | Influenza with pneumonia, virus not identified | J10 | Influenza due to other identified influenza virus | J20.5 | Acute bronchitis due to respiratory syncytial virus | U072 | COVID-19 virus not identified | J18.1 | Lobar pneumonia, unspecified |
| J02 | Acute pharyngitis | J12 | Viral pneumonia, not elsewhere classified | J11 | Influenza, virus not identified | J21.0 | Acute bronchiolitis due to respiratory syncytial virus | B34.2 | Coronavirus infection, unspecified site |  |  |
| J03 | Acute tonsillitis | J13 | Pneumonia due to Streptococcus pneumoniae |  |  | B97.4 | RSV as the cause of diseases classified to other chapters (RSV organism) | B97.2 | Coronavirus as the cause of disease classified to other chapters |  |  |
| J04 | Acute laryngitis and tracheitis | J14 | Pneumonia due to Haemophilus influenzae |  |  |  |  |  |  |  |  |
| J05 | Acute obstructive laryngitis [croup] and epiglottitis | J15 | Bacterial pneumonia, not elsewhere classified |  |  |  |  |  |  |  |  |
| J06 | Acute upper respiratory infections of multiple and unspecified sites | J16 | Pneumonia due to other infectious organisms, not elsewhere classified |  |  |  |  |  |  |  |  |
| J09 | Influenza due to identified zoonotic or pandemic influenza virus | J17 | Pneumonia in diseases classified elsewhere |  |  |  |  |  |  |  |  |
| J10 | Influenza due to other identified influenza virus | J18 | Pneumonia, organism unspecified |  |  |  |  |  |  |  |  |
| J11 | Influenza, virus not identified |  |  |  |  |  |  |  |  |  |  |
| J12 | Viral pneumonia, not elsewhere classified |  |  |  |  |  |  |  |  |  |  |
| J13 | Pneumonia due to Streptococcus pneumoniae |  |  |  |  |  |  |  |  |  |  |
| J14 | Pneumonia due to Haemophilus influenzae |  |  |  |  |  |  |  |  |  |  |
| J15 | Bacterial pneumonia, not elsewhere classified |  |  |  |  |  |  |  |  |  |  |
| J16 | Pneumonia due to other infectious organisms, not elsewhere classified |  |  |  |  |  |  |  |  |  |  |
| J17 | Pneumonia in diseases classified elsewhere |  |  |  |  |  |  |  |  |  |  |
| J18 | Pneumonia, organism unspecified |  |  |  |  |  |  |  |  |  |  |
| J20 | Acute bronchitis |  |  |  |  |  |  |  |  |  |  |
| J21 | Acute bronchiolitis |  |  |  |  |  |  |  |  |  |  |
| J22 | Unspecified acute lower respiratory infection |  |  |  |  |  |  |  |  |  |  |
| U071 | COVID- 19 virus identified |  |  |  |  |  |  |  |  |  |  |
| U072 | COVID-19 virus not identified |  |  |  |  |  |  |  |  |  |  |
| B34.2 | Coronavirus infection, unspecified site |  |  |  |  |  |  |  |  |  |  |
| B97.2 | Coronavirus as the cause of disease classified to other chapters |  |  |  |  |  |  |  |  |  |  |
| B97.4 | RSV as the cause of diseases classified to other chapters (RSV organism) |  |  |  |  |  |  |  |  |  |  |

Supplementary table 3: 28-day mortality rate following ED presentations among those who were hospitalised, by respiratory infection categories and age group

|  | ARI | | | Pneumonia | | | Influenza | | | RSV | | | COVID | | | PD | | |
| --- | --- | --- | --- | --- | --- | --- | --- | --- | --- | --- | --- | --- | --- | --- | --- | --- | --- | --- |
| Age group | Total | Death | % | Total | Death | % | Total | Death | % | Total | Death | % | Total | Death | % | Total | Death | % |
| 0-4 years | 159911 | 71 | 0.0 | 24590 | 28 | 0.1 | 6481 | 5 | 0.1 | 33810 | 12 | 0.0 | 2849 | 2 | 0.1 | 367 | 1 | 0.3 |
| 5-14 years | 23104 | 39 | 0.2 | 8592 | 24 | 0.3 | 2279 | 4 | 0.2 | 702 | 4 | 0.6 | 700 | 2 | 0.3 | 149 | 0 | 0 |
| 15-39 years | 59349 | 229 | 0.4 | 18512 | 189 | 1.0 | 4664 | 20 | 0.4 | 466 | 7 | 1.5 | 4488 | 12 | 0.3 | 1038 | 7 | 0.7 |
| 40-64 years | 76743 | 2523 | 3.3 | 49526 | 2160 | 4.4 | 6370 | 101 | 1.6 | 1292 | 44 | 3.4 | 6528 | 150 | 2.3 | 2882 | 86 | 3.0 |
| >=65 years | 200038 | 20564 | 10.3 | 146598 | 17754 | 12.1 | 14574 | 811 | 5.6 | 4141 | 238 | 5.7 | 10187 | 1339 | 13.1 | 4379 | 320 | 7.3 |
